# y-QUIT: Smoking Prevalence, Engagement, and Effectiveness of an Individualized Smoking Cessation Intervention in Youth With Severe Mental Illness

**DOI:** 10.3389/fpsyt.2018.00683

**Published:** 2018-12-14

**Authors:** Jackie Curtis, Charry Zhang, Bernadette McGuigan, Esther Pavel-Wood, Rachel Morell, Philip B. Ward, Andrew Watkins, Julia Lappin

**Affiliations:** ^1^Bondi Junction Youth Mental Health Services, South Eastern Sydney Local Health District, Sydney, NSW, Australia; ^2^Faculty of Medicine, School of Psychiatry, University of New South Wales, Sydney, NSW, Australia; ^3^Faculty of Medicine, Medical School, University of New South Wales, Sydney, NSW, Australia; ^4^Faculty of Health, University of Technology, Sydney, NSW, Australia

**Keywords:** smoking, tobacco, youth, adolescent, psychosis, first episode psychosis, at-risk for psychosis, intervention

## Abstract

**Introduction:** Young people with psychosis are six times more likely to be tobacco smokers than their gender- and age-matched peers. Smoking is a major contributor to the 15-year reduced life expectancy among people experiencing severe mental illness (SMI). There is a lack of evidence-supported interventions for smoking cessation among young people with SMI.

**Material and Methods:** The study comprised two phases and aimed to assess (i) the prevalence of smoking among a community sample of young people with psychotic illness or at high risk of developing psychosis; (ii) the proportion who engaged in the intervention; (iii) the proportion who achieved smoking cessation; and (iv) secondary smoking-related outcomes. In phase one, prevalence of smoking was assessed among young people with psychotic illness or at high risk of developing psychosis attending a community-based youth mental health service between 16/5/2017 and 16/11/2017. In phase two, over a 1-year period, individuals identified as smokers were invited to participate in a 12-week tailored smoking cessation intervention program that included pharmacological treatment, motivational interviewing, and behavioral change techniques. Those unwilling to participate in a full intervention were offered a brief intervention. Participants of the full intervention were assessed at baseline and at week 12 endpoint on: daily cigarettes smoked (self-report), exhaled CO, nicotine dependence, readiness to quit, and confidence to quit.

**Results:** In phase one, smoking prevalence was 48.2% (53 of 110) among clients of the youth mental health service. Smokers were significantly more likely to be male (*X*^2^ = 6.41 *p* = 0.009). During phase two, 41 of 61 eligible clients engaged in a smoking cessation intervention (67.2%). Effectiveness: twenty-one clients participated in a full intervention (34.4%), of whom three (14.3%) received a brief intervention initially and during engagement converted to full intervention. Twenty participants (32.8%) received a brief intervention only. Ten participants in the full intervention (47.6%) and five in the brief intervention (25%) dropped out. Six (28.6% of full intervention) reported smoking cessation verified by CO monitoring. Participants who completed the full intervention (*n* = 9) reduced number of cigarettes smoked, nicotine dependence, and exhaled CO, while readiness to quit and confidence to quit increased. Pharmacotherapy was predominantly combination NRT (*n* = 18; 85.7%), varenicline (4.8%), oral NRT only (4.8%), or none (4.8%). No adverse events were reported.

**Conclusion:** This pilot real-world study demonstrates that both screening for smoking and offering an effective smoking cessation intervention are achievable in youth experiencing or at risk of psychosis.

## Introduction

Among people experiencing severe mental illness (SMI), tobacco smoking is a modifiable risk factor for poor physical and mental health and thus a key priority for intervention. Approximately two thirds of people who experience psychotic illness smoke (1, 2). Very high smoking rates (59%) are also observed among individuals experiencing first episode psychosis (FEP), a rate six *times* that observed in age-matched peers (3). Individuals at high risk for the development of psychosis have higher tobacco use than healthy controls (4). Regular tobacco use is initiated on average (mean) 5.3 years prior to psychosis onset (3). A possible causal link between psychosis and tobacco smoking is suggested by a large meta-analysis which found overall relative risk of new onset psychotic disorders to be double that in tobacco smokers compared to non-smokers (5).

These findings suggest that daily tobacco use is associated with both increased risk for and earlier onset of psychotic illness and are thus highly relevant to populations with established FEP and those at high risk of developing psychotic illness. Within these populations, smoking among youth is a key consideration given that adolescence (12–17 years) and young adulthood (18–24 years) are critical periods in which smoking behaviors are established (6). The first cigarette is often smoked in adolescence, with tobacco experimentation generally developing into nicotine dependence before age 25 (6). Both young age and poor mental health are associated with higher levels of nicotine addiction which contribute to and sustain high smoking rates (7).

The benefits for smoking cessation and reduction in people experiencing SMI are clear, including a lowering of risk for cardiovascular and respiratory diseases and cancer—all of which are implicated in the known 10–20 year life expectancy gap (8–10). Further, smoking cessation/reduction demonstrably lowers stress levels, alleviates the financial burden associated with sustained nicotine addiction, and decreases the need for high-dose psychotropic medication, consequently lessening adverse side effects (11). Finally, long-term cessation may lead to direct clinical improvements in mental health, with improvements in anxiety and depression at levels of effect equal to or greater than those of antidepressant medication for anxiety and mood disorders (12).

Nonetheless, despite significant reductions in smoking rates among general adult populations over the past two decades, the very high smoking rates among people experiencing psychotic illness have remained almost unchanged (2, 13). It would be erroneous, however, to conclude that this reflects that people with mental health issues do not wish to stop smoking. Evidence suggests otherwise: most individuals questioned—in both mental health inpatient and community health settings—express a desire to quit (14–17) and welcome help to do so (18). Among people experiencing psychosis, 73% have attempted to quit smoking (2). Indeed, people with mental health disorders have similar or higher levels of motivation to quit when compared to the general population (19). Importantly, individuals with SMI, including young people with FEP, have poorer health literacy than healthy controls but, when shown smoking-related health warnings, they perceive them as effective (20). Nonetheless, individuals with SMI make fewer quit attempts and successful quit rates remain low (21, 22).

Understanding why individuals experiencing SMI are more likely to fail quitting is important as this will inform interventions aimed at overcoming the barriers to quit. It has been suggested that individuals experiencing SMI find quitting more difficult than do other smokers because of a range of factors including socioeconomic disadvantage, lack of familial and/or peer abstinence support and cognitive deficits (23, 24). Standard population cessation advice, which typically involves planning a quit strategy and setting a quit date, may be too cognitive an approach in some individuals with SMI, and indeed may prove counterproductive by increasing anxiety, self-stigma, and ideas of failure (25). Nonetheless, a review of smoking cessation interventions in SMI found behavioral and pharmacological interventions to be of similar effectiveness in smokers with or without SMI (26). It is unclear, however, whether these interventions would be effective in real-world settings, or be generalizable to all SMI populations, as the people with SMI who took part in those trials may have had better psychosocial function than the general SMI population (24).

Alternative interventions may be needed which are tailored to individuals experiencing SMI, or strategies employed to support their progress through cessation attempts. Intensive tailored support, provision of cessation medication, and access to peer support have each been highlighted as important elements for successful interventions (7, 27). Among youth with mental health issues, it has been suggested that cigarette smoking may act as a tool for socialization and acceptance, which should be taken into account when designing smoking cessation interventions (19). In fact, little is known about what interventions will be effective among youth smokers experiencing SMI. Among youth smokers in the general population, smoking prevalence is successfully impacted by adult-directed population-based strategies such as cigarette price increases and implementation of clean indoor air policies (28).

A recent smoking cessation trial demonstrated the feasibility of offering tailored smoking cessation interventions to adult individuals experiencing SMI. The intervention—comprising behavioral support and pharmacotherapy delivered by a specialist mental health nurse with tobacco cessation training—increased engagement with services and sustained abstinence at rates almost 3 times higher than usual care (29). Interventions such as these are yet to be trialed in youth SMI populations. The present study (y-QUIT) involved an individualized 12-week smoking cessation intervention in youth experiencing psychotic illness or at high risk of developing psychosis. The aims of the study were:

1. to measure the prevalence of self-reported smoking among a community sample of young people with FEP or at high risk of developing psychotic illness
2. to assess the proportion of individuals who engaged in the intervention
3. to assess the proportion of individuals who achieved smoking cessation, and
4. to assess secondary smoking-related outcomes.

## Materials and Methods

### Setting

This study was undertaken as part of the y-QUIT program, a local health district-funded project based in specialist early intervention in psychosis, community youth mental health (YMH) services in the South Eastern Sydney Local Health District, Sydney Australia. These comprised all YMH services across the three community mental health sites of the catchment area. The YMH services offer care to young people who have experienced first episode psychosis (FEP) or are deemed to be at ultra-high risk for development of psychosis. Inclusion criteria are age at presentation between 14 and 25 years inclusive. A 2-year program of care is offered with some individuals remaining with the service for a longer period. Ethics approval was granted by the Prince of Wales Hospital Human Research Ethics Committee [HREC ref no: 17/031 (LNR/17/POWH/50)].

### Procedures

#### Screening and Prevalence [Phase One]

Cross-sectional smoking prevalence and eligibility for the y-QUIT program was determined by administration of the Brief Assessment for Tobacco Use (Appendix 1) The Brief Assessment for Tobacco Use tool includes questions about past and current smoking (including tailor made cigarettes, roll your own, cannabis mixed with tobacco, cigars, chop chop, or waterpipe/hubbly bubbly). All clients who answered affirmatively to smoking anything in the past 30 days were identified as smokers. The screening tool was administered by the individual's caseworker or other treating clinician, the tobacco treatment specialist (BM) or the researcher-medical student (CZ). Screening was conducted either in person or by phone. Cross-sectional smoking prevalence was evaluated among all individuals who were clients of the YMH services between 16/5/2017 and 16/11/2017.

#### Identification of Eligible Participants to Engage in an Intervention [Phase Two]

All individuals who were clients of the YMH service between 16/5/2017 and 16/11/2017 [Phase One] who were identified as smokers through screening were approached to engage in the intervention. In addition, any young people newly joining the YMH services between 17/11/2017 and 15/5/2018 who were identified as smokers were approached to engage in intervention. All smokers were offered a full intervention. Those unwilling to participate in a full intervention were offered a brief intervention. Clients who denied ever smoking, or smoking in the past 12 months, were deemed ineligible.

#### Measures [Phase Two]

1. Assessment of daily cigarettes smoked by self-report.
2. Exhaled carbon monoxide (CO) measured using a Bedfont Micro Smokerlyzer (Air-met Scientific).
3. Nicotine dependence, assessed using the Heaviness of Smoking Index (HSI) (30). The HSI was developed as a test to measure nicotine dependence by using two questions from the Fagerstrom Test for Nicotine Dependence: time to first smoking in the morning and number of cigarettes per day. It uses a six-point scale calculated from the number of cigarettes smoked per day (1–10, 11–20, 21–30, 31+) and the time to first cigarette after waking (less than/equal to 5, 6–30, 31–60, and 61+ minutes). Nicotine dependence is then categorized into a three-category variable: low (0–1), medium (2–4), and high (5–6).
4. Readiness to quit by self-report (scale ranging 1–10:1 = low readiness; 10 = high readiness) (31)
5. Confidence to quit by self-report (scale ranging 1–10:1 = low confidence; 10 = high confidence) (31)

Scores on these measures were recorded by the y-QUIT tobacco treatment specialist throughout interventions on the Smoking Monitoring Form (available on request). The proportion of smokers who engaged in full and/or brief interventions was recorded by the tobacco treatment specialist. Smoking cessation by self-report was confirmed by biochemically verified CO breath test.

#### Interventions [Phase Two]

All interventions were delivered by the y-QUIT tobacco treatment specialist, a mental health nurse with additional tobacco cessation training. The tobacco treatment specialist worked closely with the multi-disciplinary YMH teams and was embedded within the Keeping the Body in Mind program which provides lifestyle interventions for these young people (32).

##### Full intervention

The intensive tobacco dependence intervention comprised an individualized 12-week program incorporating motivational interviewing, counseling support and pharmacological agents. Intensive tobacco dependence intervention involves the delivery of sessions over the phone or face to face that last longer than 10 min, with a minimum of 4 sessions. Duration of face to face appointments was typically 1 h for the first, and 30 min for subsequent sessions. Pharmacological interventions including nicotine replacement therapy (NRT) as transdermal patches, oral gum, nicotine inhaler or a combination, and varenicline, were discussed with the participant and where appropriate prescribed. NRT and prescribed smoking cessation treatments were provided as part of the y-QUIT program to participants at no cost (NRT) or cost only of prescription (varenicline). Ongoing support was provided throughout (face-to-face and by phone) and there was regular monitoring of mental state changes and adverse side effects through clinical assessment by the treatment specialist in conjunction with the clinical team. Psychotropic medications were monitored, and doses adjusted by the treating psychiatrist as required. At baseline and at 4-weekly intervals thereafter terminating at week 12, participants completed the following smoking-related measures: daily cigarettes smoked, exhaled CO, nicotine dependence, readiness to quit, and confidence to quit.

##### Brief intervention

Brief interventions typically comprised 1–2 sessions delivered face to face or by telephone. The goal of a brief intervention was to initiate change in behavior, utilizing the 5A's model (33). A person's smoking risk level was assessed using a validated CO monitor. Motivational interviewing, counseling, and measurement of exhaled CO were used to engage the individual in a discussion about readiness to change smoking behavior. Pharmacotherapy was available as NRT. Participants were encouraged to convert to the full intervention. Harm reduction strategies were offered to those who chose to continue to smoke.

### Outcomes [Phase Two]

The primary outcome was smoking cessation by self-report at 12-week endpoint in the full intervention with confirmation of abstinence by exhaled CO measure of ≤ 4 ppm. Secondary outcomes (number of cigarettes smoked per day; exhaled CO; nicotine dependence; readiness to quit; confidence to quit) and pharmacotherapy used were recorded at 12-week endpoint for all those who completed the full intervention.

### Statistical Analysis

Statistical analyses were conducted with IBM SPSS Statistics Version 24 (IBM Corp 2016). For continuous variables, means and standard deviations (SD) or medians and interquartile ranges were calculated. For tests of linear trend for categorical variables chi square was calculated. Secondary smoking-related outcomes were analyzed descriptively using mean (SD) or median (range).

## Results

### Prevalence of Smoking [Phase One]

The prevalence of self-reported smoking among young people with FEP or at high risk of developing psychotic illness was 48.2% (53 of 110). Smokers were significantly more likely to be male (*X*^2^ = 6.41 *p* = 0.009; Table 1).

**Table 1:** Comparison of demographic characteristics between smokers and non-smokers among youth engaged with a community early intervention in psychosis service.

	**Smokers (*n* = 53)**	**Non-smokers (*n* = 57)**	
Male (*n*)	41	31	*X*^2^ = 6.41 *p* = 0.009
Female (*n*)	12	26	
Age (median, range)	21 (18–27)	22 (16–26)	*t* = 0.309 *p* = 0.76
Age (mean, SD)	21.3 (2.3)	21.5 (2.3)	

### Engagement: Proportion of Individuals Who Engaged in an Intervention [Phase Two]

During Phase Two, of the 61 clients offered y-QUIT, 41 engaged in a smoking cessation intervention (67.2%). Twenty-one clients participated in a full intervention (34.4%), of whom three (14.3%) received a brief intervention initially and during engagement converted to full intervention. The mean number of sessions for full intervention was 5.1 (SD = 3.4; median = 6 (range 15). Twenty participants (32.8%) received a brief intervention only. Sixteen individuals (26.2%) declined participation in any intervention, and a further four clients (6.6%) were unable to participate due to discharge from the service. The vast majority of the individuals who participated in the full (90.5%), and brief intervention (80.0%), were male (Table 2). Mean ages were 22.1 (2.0) and 20.6 (2.0) years, respectively. Ten participants in the full intervention (47.6%) dropped out and two (9.6%) were discharged from the YMH service before completion.

**Table 2:** Demographic characteristics, quit prevalence, and pharmacotherapy received among smokers who received full or brief intervention.

	**Full intervention (*n* = 21)**	**Brief intervention (*n* = 20)**	**All participants (*n* = 41)**
Age (median, range)	22 (19–25)	20 (18–25)	21 (18–25)
Age (mean, SD)	22.1 (2.0)	20.6 (2.0)	21.3 (2.1)
**GENDER**			
Male (n)	19	16	35
Female (n)	2	4	6
Quit smoking (n, %)	6 (28.6)	0	6 (14.6)
**PHARMACOTHERAPY (*****n*****, %)**			
Oral NRT only	1 (4.8)	2 (10.0)	3 (7.3)
Combination NRT	18 (85.7)	4 (20.0)	22 (53.7)
Varenicline	1 (4.8)	–	1 (2.4)
None	1 (4.8)	14 (70.0)	15 (36.6)

### Effectiveness: Proportion of Individuals Who Achieved Smoking Cessation [Phase Two]

Six individuals (28.6% of full intervention; 14.6% of all interventions) reported smoking cessation (verified by CO monitoring) at completion of the full intervention (Table 2).

### Effectiveness: Secondary Smoking-Related Outcomes [Phase Two]

A further 3 participants (14.3% of full intervention) completed the full intervention and reduced the number of cigarettes smoked each day. As a group, participants who completed the full intervention (*n* = 9) reduced number of cigarettes smoked, nicotine dependence, and exhaled CO (Table 3). Both readiness to quit and confidence to quit increased (Table 3). Pharmacotherapy in the full intervention was predominantly combination NRT (*n* = 18; 85.7%), with one client each prescribed varenicline (4.8%), oral NRT only (4.8%) or no pharmacotherapy (4.8%; Table 2). No adverse events were reported.

**Table 3:** Smoking-related measures at baseline in clients who received the full intervention (*n* = 21), and at baseline and 12-week endpoint in those who completed the full intervention (*n* = 9).

	**Baseline (*n* = 21)**	**Baseline (completers; *n* = 9)**	**Endpoint (completers; *n* = 9)**
Number of cigarettes (mean, SD)	15.3 (9.8)	14.2 (11.8)	1.1 (1.9)
Number of cigarettes (median, IQR)	12.5 (0–35)	12.5 (0–35)	0 (0–6)
Heaviness smoking index (median, IQR)	3 (0–6)	3 (0–5)	0 (0–3)
Exhaled carbon monoxide (ppm; mean, SD)	15.8 (7.2)	14.8 (7.7)	5.9 (7.3)
Exhaled carbon monoxide (ppm; median, IQR)	16 (3–28)	15 (3–24)	3 (2–25)
Readiness to quit score (median, IQR)	7 (1–10)	7 (3–10)	10 (3–10)
Confidence to quit score (median, IQR)	7 (1–10)	7 (6–10)	10 (4–10)

## Discussion

### y-QUIT Smoking Cessation Intervention

To our knowledge, the engagement of youth with severe mental illness and the effectiveness of tailored smoking cessation interventions in this population have never previously been reported. This real-world study demonstrated that the delivery of an individualized smoking cessation intervention is both achievable and effective in a community youth mental health service.

#### Smoking Prevalence Among Youth With SMI

The prevalence of self-reported smoking of 48.2% was somewhat lower than typical rates found in general SMI populations, but is consistent with the estimated prevalence rate of 59% in individuals with FEP (3). Smokers were significantly more likely to be male, even accounting for the greater proportion of males making up this population of FEP and at-risk for psychosis, in this younger-age demographic (≤ 25 years at presentation).

#### Engagement With Intervention

Uptake rates of 67.2% in either a full or brief smoking cessation intervention in this sample confirm that youth with SMI have an interest in quitting tobacco smoking. Indeed, just over a quarter of YMH service clients declined participation in either intervention. Despite this high interest however, the full intervention was engaged in by just over half of these individuals. Of note, three individuals initially offered a brief intervention converted to the full intervention once engaged. One further young person offered brief intervention was discharged from the YMH service before completing the brief intervention as he had elected to trial an inpatient rehabilitation for comorbid cannabis use. He subsequently reported that he had quit both tobacco and cannabis. While this quit was not included in the numbers reported here, it is evidence of another positive impact of the y-QUIT program on smoking behavior in young people. This provides support for offering a brief intervention in order to provide a gateway to sustaining interest in and garnering commitment to engagement in a full intervention. A notable proportion of individuals dropped out: almost half of those who initiated a full intervention. Some individuals who initially declined engaging with the full intervention or who dropped out became engaged or re-engaged with the tobacco treatment specialist after the study period had completed. Previous studies investigating smoking cessation interventions in people with SMI have similarly noted high dropout rates and that both smoking cessation and smoking reduction are more likely among those individuals who engage fully with the intervention (34). This is consistent with knowledge that tobacco dependence is a chronic condition and that repeated attempts are typically required to stop smoking successfully (29, 35).

#### Successful Quitting

Approximately one third of individuals who participated in the full intervention reported smoking cessation at the 12-week endpoint. This is a highly encouraging outcome and suggests that this intervention is effective in youth with SMI, at least in the short-term. Nonetheless, two-thirds of participants were unsuccessful in quitting. This, together with the high drop-out rates, also raises the question whether current evidence and/or service user feedback might enhance the current approach to make the interventions more acceptable. Future interventions may need to incorporate recognition of these factors as obstacles to quitting, and perhaps discuss strategies of harm minimization (smoking reduction, use of NRT) as an initial alternative goal to quitting.

#### Secondary Smoking-Related Outcomes

All participants who completed the full intervention reduced daily number of cigarettes smoked, nicotine dependence, and exhaled CO. This is important particularly in the youth population, where there is evidence of a dose-response relationship between increased number of cigarettes smoked and risk for psychosis (36–38). In a large 15-year follow-up study of psychosis risk and its relationship to tobacco use in adolescence, smoking 10 or more cigarettes daily was associated with a significantly increased risk for psychosis compared to not smoking, while light smoking (1–9 cigarettes daily) was not (38). This provides an additional argument to support harm minimization of smoking in youth with SMI, and, if tobacco smoking were causal in increasing psychosis risk, is particularly relevant to those at high-risk for psychosis. Young people reported increased readiness and confidence to quit on completion of the full intervention. While both measures showed a range of scores across participants, baseline scores were high in the majority. This concords with previous evidence that smokers with SMI have the desire to quit, but may require additional assistance in order to successfully do so (19). Finally, pharmacotherapy used was predominantly combination NRT but a range of approaches—including use of varenicline or alternatively use of no pharmaceutical agent—was applied, in keeping with the focus on individualized care.

### Limitations of y-QUIT

The present study presents preliminary 12-week outcomes only and cannot speak to long-term effectiveness of this intervention. Previous smoking cessation studies including the SCIMITAR trial in SMI adults have assessed smoking cessation at 1 year following randomization to intervention (29). Screening for smoking status was only conducted routinely during Phase One, that is, the first 6 months of the 12-month program. During the latter 6 months, an additional eight young people who were identified as smokers were offered the program. It is, however, possible that among all young people newly entering the YMH services in that 6-month period there may have been additional smokers who were not identified in the absence of screening. Lastly, brief interventions did not routinely assess smoking-related measures or include follow-up evaluation to assess their effectiveness in increasing desire, confidence, and readiness to quit. This is an important area for future development, particularly given the potential that brief interventions act as segue into full interventions.

### Implications of y-QUIT for Future Interventions

Internationally, there is growing recognition of the need to integrate smoking cessation into the treatment of people experiencing SMI and the need to adapt programs developed in the general population to address the specific needs of people living with mental illness (24, 39). There remains an overwhelming need for smoking to be addressed more adequately in mental health services. A combination of culture change, increased accessibility to intervention programs (both pharmacological and non-pharmacological), and staff training are necessary to address the life expectancy gap and inequality experienced by youth with SMI. Future studies which include varenicline as part of a routine intervention should also be considered and may increase the quit rates, given recent evidence indicating the efficacy and relative safety of this treatment in SMI populations (40).

y-QUIT incorporated all elements recommended as necessary to reduce the very high rates of smoking amongst this population, namely training in brief interventions, motivational interviewing, and pharmacological support (41). That delivery of all interventions was by a mental health nurse with additional tobacco cessation training demonstrates that this model is immediately translatable to community mental health settings where there is sufficient funding and support from clinicians and managers to do so.

### Conclusion

Smokers experiencing SMI are a priority target group for smoking cessation interventions. The need to provide smoking cessation to youth with SMI is all the more urgent, as successful quitting at as early a stage as possible will optimally reduce risk for smoking-related disease and life expectancy shortening. This first-of-its-kind real-world smoking cessation program demonstrates that both screening for smoking and offering an effective smoking cessation intervention is acceptable and effective in youth mental health services. Individuals with SMI should be asked about smoking and should be provided with smoking cessation interventions. The very high rates of tobacco smoking in this population, and the failure of public health measures to have had significant impact demand further urgent work in tailoring interventions effective in this priority group.

## Author Contributions

JC, EP-W, AW, and PW conceived the initial project. AW and BM designed the intervention. CZ, BM, RM, and AW contributed to data collection. EP-W and CZ conducted the background literature review. JL, CZ and RM conducted data analysis. JL and JC wrote the first draft of the manuscript. All authors contributed to and approved the final manuscript.

### Conflict of Interest Statement

The authors declare that the research was conducted in the absence of any commercial or financial relationships that could be construed as a potential conflict of interest.
